# Malaria case investigation with reactive focal testing and treatment: operational feasibility and lessons learned from low and moderate transmission areas in Amhara Region, Ethiopia

**DOI:** 10.1186/s12936-018-2587-8

**Published:** 2018-12-04

**Authors:** Pooja Bansil, Asnakew K. Yeshiwondim, Caterina Guinovart, Belendia Serda, Callie Scott, Berhane H. Tesfay, Adem Agmas, Belay Bezabih, Melkamu T. Zeleke, Girma S. Guesses, Asmamaw L. Ayenew, Worku M. Workie, Duncan Earle, Rick W. Steketee, Asefaw Getachew

**Affiliations:** 1 PATH Malaria Control and Elimination Partnership in Africa (MACEPA), 2201 Westlake Avenue, Suite 200, Seattle, WA 98121 USA; 2 PATH Malaria Control and Elimination Partnership in Africa (MACEPA), Addis Ababa, Ethiopia; 3 Amhara National Regional State Health Bureau, Bahir Dar, Ethiopia

**Keywords:** Malaria, Surveillance, Elimination, Case Investigation, Ethiopia

## Abstract

**Background:**

When malaria transmission is very low, investigation of passively detected malaria cases and reactive focal testing and treatment (FTAT) in the case and neighbouring households can identify and contain the source and spread of infections.

**Methods:**

Case investigation with reactive FTAT for malaria was implemented in 10 villages in Amhara Region, Ethiopia during the 2014/2015 malaria transmission season. Intervention villages were purposively selected based on the incidence of passively detected *Plasmodium falciparum* and mixed infections (*P. falciparum* and *Plasmodium vivax*) during the 2013 transmission season. A passively detected *P. falciparum* or mixed index case triggered an investigation that targeted the index case household and the closest 10 neighbouring households in a 100-m radius. All consenting household members received a rapid diagnostic test (RDT) and RDT-positive individuals received artemether–lumefantrine (*P. falciparum*, mixed) or chloroquine (*P. vivax*).

**Results:**

From October 2014 to February 2015, 407 *P. falciparum* or mixed index cases (approximately 6.5 per 1000 population) were passively detected. Of these, 220 (54.1%) were investigated, of which 87.3% were male, 61.8% were age 20–39 years [median age: 27 years (range 1–90)], and 58.6% spent ≥ 1 night away from home in the past month (ranging from 0.0 to 94.1% by village). Among the 4077 residents in the 914 households investigated, 3243 (79.5%) received an RDT and 127 (3.9%) were RDT-positive (2.2% *P. falciparum*, 0.5% *P. vivax*, 1.2% mixed). Three epidemiological patterns were found. In six villages, there were almost no cases, with less than 10 index and secondary cases. In three villages, most index cases had a history of travel (> 62%), but there were a small number of secondary cases (< 10). Lastly, in one village none of the index cases had a history of recent travel and there was a large number of secondary cases (n = 105).

**Conclusions:**

Three types of malaria transmission patterns were observed: (1) low importation and low local transmission; (2) high importation and low local transmission; and, (3) low importation and high local transmission. To achieve malaria elimination in Amhara Region, intervention strategies targeting these different patterns of transmission and population movement are required.

**Electronic supplementary material:**

The online version of this article (10.1186/s12936-018-2587-8) contains supplementary material, which is available to authorized users.

## Background

Malaria is one of the leading public health challenges in Ethiopia with an estimated 68% of the population living in malaria-risk areas [1]. In Ethiopia, both *Plasmodium falciparum* and *Plasmodium vivax* parasites are transmitted, and malaria transmission is generally unstable with seasonal variations that are largely determined by altitude, climate and human settlement patterns [1]. In 2005, Ethiopia initiated a substantial scale up of malaria prevention tools and case management interventions that resulted in a significant decline in the number of cases and deaths associated with malaria [2].

The Federal Ministry of Health (FMOH) of Ethiopia aims to achieve malaria elimination in more than 239 districts by 2020 and the Ethiopia Malaria National Strategic Plan includes, as a priority action point for malaria elimination, the establishment of a system for case and foci investigation and classification [1]. The Amhara Malaria Elimination Demonstration Project, a collaborative effort between the Malaria Control and Elimination Partnership in Africa (MACEPA) at PATH, the Carter Center, the FMOH and the Amhara National Regional State Health Bureau (ANRSHB), was established to support sub-national malaria elimination through the pilot implementation and evaluation of several interventions to rapidly reduce malaria transmission in the Amhara Region and inform regional scale up.

Case investigation with reactive focal test and treat (FTAT) is an intervention to identify malaria infections in a community, particularly where transmission is focal and individuals living close to a symptomatic case are at a higher risk of being infected. A malaria case passively detected at a health facility or in the community (an index case) triggers an investigation in the index case household and neighbouring households to identify other malaria cases. If there is capacity to establish a good passive case detection system and conduct case investigation and FTAT for all index cases, this strategy could potentially reduce malaria infections in the community and thus contribute to decreased transmission and progress towards elimination [3–5]. The objective of this intervention was to inform the FMOH’s strategy for malaria elimination by evaluating the feasibility of focal malaria parasite clearance strategies and describing patterns of importation and transmission in 10 villages in Amhara Region.

## Methods

### Study area

Amhara Region, located in northwestern Ethiopia, had an overall estimated malaria parasite prevalence in children 6–59 months old in 2015 of 1.1% detected by microscopy, of which 78.6% was attributable to *P. falciparum* and 21.4% to *P. vivax* [6]. Malaria transmission in Amhara usually peaks between September and November, but differs substantially by geography due to variations in altitude, temperature and annual rainfall, resulting in unique eco-epidemiological malaria risk and transmission zones.

The Amhara Malaria Elimination Demonstration Project activities span these diverse zones and encompass a total of 213 health posts (within 209 villages) in eight districts. Accounting for population, altitude and passively detected rapid diagnostic test (RDT)-confirmed *P. falciparum* and mixed malaria cases during the 2013 peak malaria season, villages were stratified into the following strata: (1) no transmission (0 *P. falciparum*/mixed malaria cases per 1000 population per week); (2) very low transmission (> 0 and < 0.3 cases per 1000 population per week); (3) low transmission and high altitude (≥ 0.3 and < 1 case per 1000 population per week and an altitude ≥ 2000 m); (4) low transmission and low altitude (≥ 0.3 and < 1 case per 1000 population per week and an altitude < 2000 m); and, (5) moderate transmission (≥ 1 case per 1000 population per week).

**Fig. 1:**
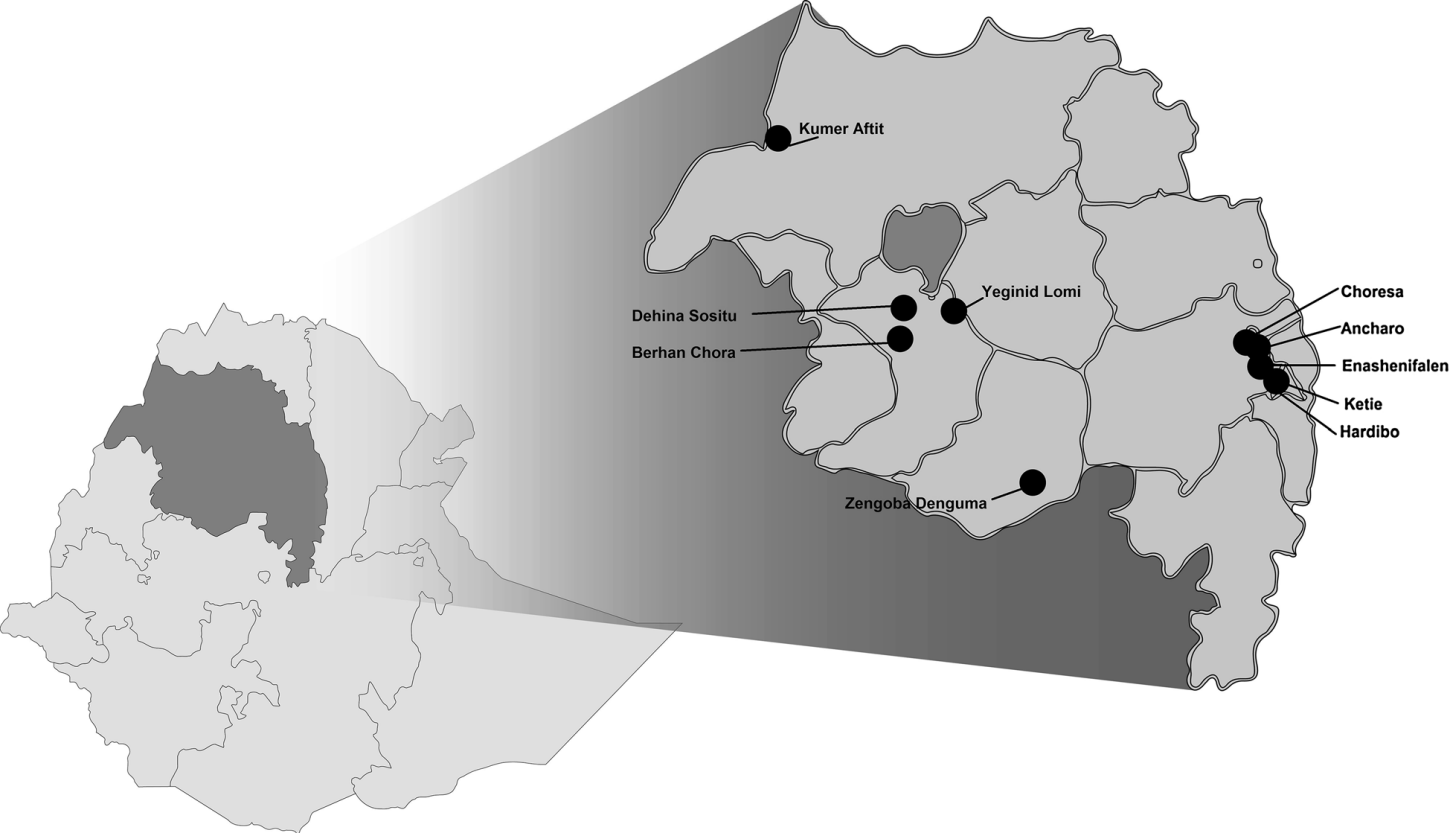
Study area for case investigation with focal test and treat, Amhara Region, Ethiopia. Map of Amhara, Ethiopia showing the location of the health posts serving the 10 intervention villages where case investigation with reactive focal test and treat intervention was implemented

Samples of two villages from each transmission stratum, resulting in a total of 10 villages, were purposively selected to participate in the case investigation with reactive FTAT intervention. Following rapid field data validation results, villages were selected on the basis of health post malaria data completeness and accuracy, and absence of a health centre in the area. The selected villages were Ancharo, Berhan Chora, Choresa, Dehina Sositu, Enashenifalen, Hardibo, Kumar Aftit, Ketie, Yeginid Lomi and Zengoba Denguma (Fig. 1) and covered approximately a total population of 63,000 [7]. All households in the selected villages were censused and mapped before starting the intervention.

### Intervention design

In the six villages with the highest malaria incidence, a mass test-and-treat (MTAT) intervention, where the entire population in each village was tested with RDTs and those that tested positive were treated, was conducted in September 2014, prior to the case investigation and reactive FTAT intervention as described elsewhere [8]. Starting in October 2014, a pilot case investigation with FTAT was implemented in all 10 intervention health posts, where according to national guidelines, all patients who presented to a health post or to a health extension worker (HEW) during community outreach with fever, history of fever or other malaria-compatible symptoms, received a CareStart™ Malaria HRP2/pLDH (Pf/PAN) Combo (ACCESSBIO) RDT and, if positive, received anti-malarial treatment [9].

Upon diagnosis of a *P. falciparum* or mixed RDT-confirmed malaria index case, a surveillance assistant (SA) was notified to review the health post register, gather additional information about the case (age, gender, address) in a standardized questionnaire and determine whether it was possible to conduct a case investigation with FTAT. Index cases were not investigated if they resided outside the health post catchment area, had insufficient information to identify the case’s household, were visitors staying fewer than 21 days in the village, or resided in a recently investigated household.

For eligible index cases, SA teams located the index case household and conducted a reactive FTAT investigation to identify additional secondary infections in the index case household and as many as 10 closest neighbouring households within a radius of approximately 100 m. At each consented household, teams administered household questionnaires to collect information on the geographic location of the household, the number of household members, mosquito net ownership, and receipt of indoor residual spraying (IRS) in the past 12 months. For all household members aged 6 months and older who provided informed oral consent and/or assent, SAs measured axillary temperature and performed an CareStart^TM^ Malaria HRP2/pLDH(Pf/PAN) Combo (ACCESSBIO) RDT test. Teams also administered individual questionnaires to capture information on socio-demographics (age, gender, education, occupation) and individual risk factors for malaria [travel history (spent one or more nights away from home, outside their current residence, in past month), mosquito net usage, fever]. Any participant with a positive RDT was administered anti-malarial treatment per national guidelines (artemether–lumefantrine (AL) for *P. falciparum* or mixed infection and chloroquine for *P. vivax*). Follow-up visits were conducted among all FTAT participants receiving AL to evaluate adherence and adverse events for 3–7 days after administration of the first dose. In Dehina Sositu and Yeginid Lomi, the follow-up visits also included collection of a blood smear to evaluate parasitaemia clearance from day 7 to day 13 after administration of the first dose. Giemsa-stained thick and thin films were prepared on the same smear and were read by microscopy at the regional Bahir Dar laboratory.

This study protocol was reviewed and approved by the Amhara National Regional State Health Bureau Research Ethics Review Committee, and the study received a non-research determination from PATH.

### Data collection and analysis

Individual and household questionnaires were developed and customized using Open Data Kit (ODK); data were collected using smartphones. Depending on Internet connectivity, data from completed questionnaires were uploaded directly from the smartphones or manually downloaded to a secure data repository. This study focuses on case investigations and reactive FTAT visits conducted between 20 October, 2014 and 28 February, 2015.

Statistical analyses were conducted in Stata 13.1 (Statacorp, College Station, TX, USA). A descriptive analysis of index cases, households visited and individuals tested during the FTAT was conducted for each village. In addition, descriptive statistics (mean and range) were estimated for the following operational considerations overall and by village: index cases diagnosed per week, index cases investigated each week, mean number of households investigated per index case, and time (days) between diagnosis and initiation of case investigation and time to complete an investigation.

## Results

**Fig. 2:**
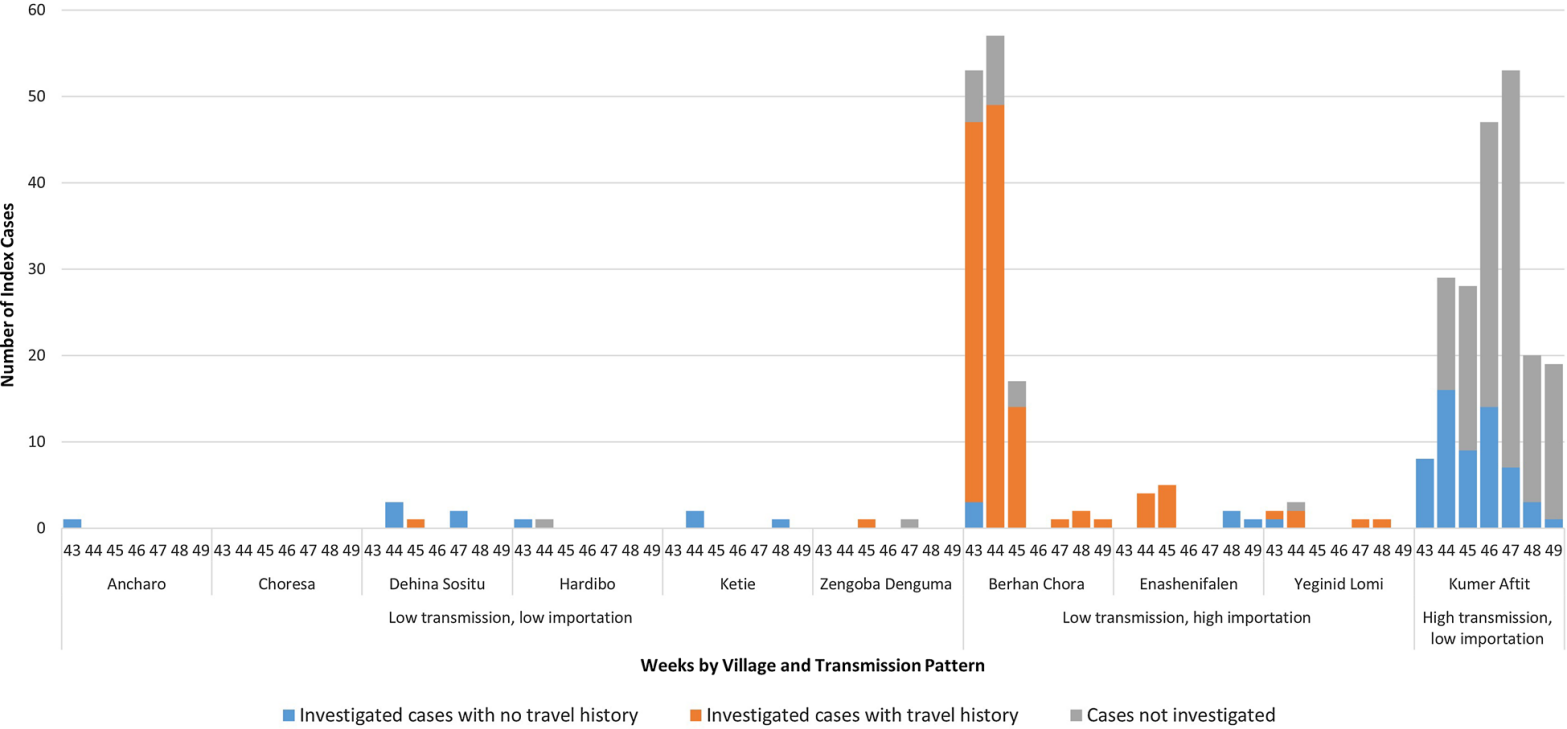
Number of index cases during the peak malaria transmission (weeks 43–49), by investigation status, case travel history and village, Amhara Region, Ethiopia. Bar chart showing the number of index cases by investigation status and case travel history for each of the 10 intervention villages

During the study period in the 10 intervention villages, a total of 407 passively detected *P. falciparum*/mixed cases were identified at the health posts (summary clinical case rate of 6.5 per 1000 population for this peak transmission season interval). The weekly number of index cases reported during the peak malaria transmission season (weeks 43–49) is illustrated in Fig. 2. The total number of passive cases detected by village in this interval ranged from 1 to 228 and the highest number of cases were observed in two villages accounting for 89% of all cases: Kumer Aftit (n = 228) and Berhan Chora (n = 134). In Berhan Chora with a mean of 20.4 cases diagnosed per week, where 89% of the investigated index cases had travelled in the past month, the FTAT procedures were modified (on 1 November, 2014) because it was not possible to complete FTAT for every index case. After this date, if an index case in Berhan Chora had a travel history, only the index case household was visited. Kumer Aftit had 8.5 cases diagnosed per week and none of the investigated index cases reported recent travel. However, a large number of index cases in Kumer Aftit were not investigated because, after several weeks of investigation, the majority (64.7%) of cases eligible for investigation resided in households that had been included in a prior investigation.

Of the 407 index cases identified, 220 (54%) were investigated. For the 187 (46%) cases not investigated, reasons included: no one could be found at home at the index case household during the FTAT investigation (0.6%); the index case was a visitor who spent fewer than 21 days in the village (34.2%); the household had been previously investigated as part of another index case’s investigation (63.1%); or due to other reasons (2.1%). Of the 187 index cases not investigated, 75.9% were males, the largest group were 20–39 years old (41.2%), and 92.2% were from Kumar Aftit.

A total of 873 individuals from 220 index case households and 2983 from 694 neighbouring households were visited (Table 1). Of these, 772 (88.4%) individuals from index case households and 2471 (82.8%) from neighbouring households consented and were tested with a malaria RDT. The overall RDT-positivity rate was 4.8% (37/772) and 3.6% (90/2471) in index case and neighbouring households, respectively.

**Table 1:** Study profile of index cases and FTAT investigation, by transmission and importation category

	Low transmission, low importation villages 6 villages: Ancharo, Choresa, Dehina Sositu, Hardibo, Ketie, Zengoba Denguma	Low transmission, high importation villages 3 villages: Berhan Chora*; Enashenifalen; Yeginid Lomi	High transmission, low importation village 1 village: Kumar Aftit*	Total 10 villages
2015 population estimates	34,294	23,684	3666	61,644
Index cases				
Passively detected *P. falciparum*/mixed, n	23	156	228	407
Investigated, n (%)	18 (78.3)	141 (90.4)	61 (26.8)	220 (54.1)
Investigated cases with travel history in past 30 days, n (%)	2 (11)	126 (89.4)	0 (0)	128 (58.2)
Investigated cases with no travel history in past 30 days, n (%)	16 (89.0)	15 (10.6)	61 (100.0)	92 (41.8)
Interval parasite index (per 1000 population in the high transmission season)	0.67	6.6	62.2	6.6
FTAT investigation				
Index case HH				
Individuals in HHs, n (excluding index cases)	74	529	270	873
Individuals tested, n (%)	66 (89.2)	503 (95.1)	203 (75.2)	772 (88.4)
RDT positivity, n (%)	5 (7.6)	7 (1.4)	25 (12.3)	37 (4.8)
Neighbouring HH				
HHs visited, n	112	254	328	694
Individuals in HHs, n	529	1113	1341	2983
Individuals tested, n (%)	467 (88.3)	1002 (90.0)	1002 (74.7)	2471 (82.8)
RDT positivity, n (%)	2 (0.4)**	8 (0.8)	80 (8.0)**	90 (3.6)

FTAT, focal test and treat; HH, household

* In Berhan Chora and Kumer Aftit, the FTAT procedures were modified on 1 November, 2014 because it was not possible to complete case investigations for every index case. If the index case had a travel history, only the index case household was visited for the case investigation rather than the index case household plus up to 10 neighbouring households

** Significant *P* value comparing RDT positivity in Index HH and Neighbouring HH (*P *< 0.05)

The transmission season parasite index varied across the 10 villages by more than 100-fold and the RDT-positivity rate among investigated residents in index case households ranged from 0 to 33.3%, and among neighbouring households ranged from 0 to 8.0% (Additional file 1). The proportion of index cases with a history of travel to a higher malaria transmission area in the past 30 days varied from 0 to 94% across the sites (Additional file 1). Based on village transmission rates and patterns of travel history among investigated index cases, three distinct village patterns were identified: (1) low transmission and low importation; (2) low transmission and high importation; and, (3) higher transmission and low importation. Subsequent analyses are presented for these specific groupings.

### Low transmission and low importation (6 villages)

In the villages of Ancharo, Choresa, Dehina Sositu, Hardibo, Ketie, and Zengoba Denguma, with a combined population of approximately 34,300 people, there were 23 index cases (interval parasite index = 0.67 cases/1000 population) among which 87% had no travel history in the previous month. Among 74 individuals in these index case households, 89% were tested and 5 additional RDT-positive cases (3 *P. falciparum* and 2 mixed) were found (7.5% RDT-positivity rate). Among 529 investigated individuals in neighbouring households, 88% were tested and 2 additional RDT-positive cases (both *P. falciparum*) were found (0.4% RDT-positivity rate; significantly lower than in index case households, p < 0.01) (Table 1). Given the small number of investigated index cases (n = 18) and secondary cases (n = 7), no significant socio-behavioural differences were found between these groups (Table 2).

**Table 2:** Number and per cent distribution of socio-demographic characteristics and malaria-risk factors of index and secondary cases detected during the FTAT, Amhara Region, Ethiopia

	Low transmission, low importation		Low transmission, high importation		High transmission, low importation	
	Index cases	Secondary cases	Index cases	Secondary cases	Index cases	Secondary cases
	*P. falciparum* or mixed (n = 18) n (%)	*P. falciparum, P. vivax* or mixed (n = 7) n (%)	*P. falciparum or mixed* (n = 141) n (%)	*P. falciparum, P. vivax* or mixed (n = 15) n (%)	*P. falciparum or mixed* (n = 61) n (%)	*P. falciparum, P. vivax* or mixed (n = 105) n (%)
Gender						
Female	6 (33.3)	4 (57.1)	5 (3.6)	3 (20.0)**	16 (26.2)	40 (38.1)
Male	12 (66.7)	3 (42.9)	136 (96.4)	12 (80.0)	45 (73.8)	65 (61.9)
Age (years)						
< 5	1 (5.6)	1 (14.3)	0 (0)	3 (20.0)**	1 (1.6)	10 (9.5)**
5–9	1 (5.6)	2 (28.6)	1 (0.7)	1 (6.7)	5 (8.2)	27 (25.7)
10–19	6 (33.3)	1 (14.3)	13 (9.2)	1 (6.7)	26 (42.6)	35 (33.3)
20–39	7 (38.9)	2 (28.6)	111 (78.7)	9 (60.0)	17 (27.9)	31 (29.5)
40–59	1 (5.6)	1 (14.3)	16 (11.4)	1 (6.7)	10 (16.4)	2 (1.9)
≥ 60	2 (11.1)	0 (0)	0 (0)	0 (0)	2 (3.3)	0 (0)
Occupation						
None	2 (11.1)	1 (14.3)	0 (0)	0 (0)**	10 (16.4)	15 (14.3)**
Child < 10 years old	2 (11.1)	3 (42.7)	1 (0.7)	4 (26.7)	6 (9.3)	37 (35.2)
Farmer	11 (61.1)	3 (42.7)	132 (93.6)	11 (73.3)	32 (52.5)	27 (25.7)
Other	3 (16.7)	0 (0)	8 (5.7)	0 (0)	13 (21.3)	26 (24.8)
Education						
None	11 (61.1)	3 (42.7)	95 (67.4)	8 (53.3)**	39 (63.9)	31 (29.5)**
Child < 10 years old	2 (11.1)	3 (42.7)	1 (0.7)	0 (0)	6 (8.8)	37 (35.2)
Primary School	5 (27.8)	0 (0)	33 (27.0)	3 (20.0)	13 (21.3)	36 (34.3)
Secondary or higher	0 (0)	1 (14.3)	7 (5.0)	4 (26.7)	3 (4.9)	1 (1.0)
Slept under a bed net past night						
No	5 (27.8)	3 (42.9)	86 (61.0)	5 (33.3)*	9 (14.8)	20 (19.1)
Yes	13 (72.2)	4 (57.1)	55 (39.0)	10 (66.7)	52 (85.3)	85 (80.9)
Travel history in past 30 days						
No	16 (88.9)	7 (100.0)	15 (10.6)	12 (80.0)**	61 (100.0)	105 (100.0)
Yes	2 (11.1)	0 (0)	126 (89.4)	3 (20.0)	0 (0)	0 (0)
HH received IRS in past 12 months						
No	13 (72.2)	5 (71.4)	22 (15.6)	6 (40.0)*	61 (100.0)	105 (100.0)
Yes	5 (27.8)	2 (28.6)	119 (84.4)	9 (60.0)	0 (0)	0 (0)
Received anti-malarial treatment in past 2 weeks						
No	–	6 (85.7)	–	15 (100.0)	–	104 (99.1)
Yes	–	1 (14.3)	–	0 (0)	–	1 (0.9)
Measured fever at time of diagnosis						
No	15 (83.3)	7 (100.0)	76 (53.9)	15 (100.0)**	35 (57.4)	97 (92.4)**
Yes	3 (16.7)	0 (0)	65 (46.1)	0 (0)	26 (42.6)	8 (7.6)
Travel history in past 30 days						
No	16 (88.9)	7 (100.0)	139 (98.6)	7 (46.7)**	61 (100.0)	105 (100.0)
Yes	2 (11.1)	0 (0)	2 (1.4)	8 (53.3)	0 (0)	0 (0)

–, not ascertained at the time of diagnosis; HH, household; IRS, indoor residual spraying

* Significant Chi square P value between 0.01 and < 0.05 comparing demographic characteristics and malaria risk factors between index *P. falciparum* or mixed cases and secondary *P. falciparum, P. vivax* or mixed cases, within a transmission pattern. For categorical variables with multiple categories, the Chi square P value refers to the overall comparison across all categories.

** Significant Chi square P value < 0.01 comparing demographic characteristics and malaria risk factors between index *P. falciparum* or mixed cases and secondary *P. falciparum, P. vivax* or mixed cases, within a transmission pattern. For categorical variables with multiple categories, the Chi square P value refers to the overall comparison across all categories.

### Low transmission and high importation (3 villages)

In the villages of Berhan Chora, Enashenifalen and Yeginid Lomi, with a combined population of approximately 23,700 people, there were 156 index cases (interval parasite index = 6.6 cases/1000 population) among which 89% reported travel in the previous month. Among 529 individuals in these index case households, 95% were tested and 7 additional RDT + cases (5 *P. falciparum*, 1 *P. vivax* and 2 mixed) were found (1.4% RDT-positivity rate). Among 1113 individuals in neighbouring households, 90% were tested and 8 additional RDT + cases (4 *P. falciparum*, 3 *P. vivax* and 1 mixed) were found (0.8% RDT-positivity rate) (Table 1). When comparing the 141 index cases with the 15 secondary cases, the following significant differences were found: index cases were more likely to be male (96 vs 80%), between the ages of 10–39 (88 vs 67%), have no education (67 vs 53%), did farm-related work (94 vs 73%, especially work as migrant labourers in large farms), report not sleeping under a mosquito net the previous night (61 vs 33%), had no measured fever at the time of diagnosis (54 vs 100%), have spent one or more nights away from home in the past month (89 vs 20%) and lived in a household with other individuals having travelled (99 vs 47%) (Table 2).

### Higher transmission and low importation (1 village)

In the village of Kumar Aftit, with an estimated population of 3666 people, 228 index cases were identified (interval parasite index = 62.2 cases/1000 population) among which none reported travel in the previous month. Among 270 individuals in these index case households, 75% were tested and 25 additional RDT + cases (16 *P. falciparum*, 2 *P. vivax* and 7 mixed) were found (12.3% RDT-positivity rate). Among 1341 individuals in neighbouring households, 75% were tested and 80 additional RDT + cases (42 *P. falciparum*, 11 *P. vivax* and 27 mixed) were found (8% RDT-positivity rate, significantly lower than in index case households, p = 0.046). Significant differences between the 61 index cases investigated and the 105 secondary cases identified included index cases being more likely to be age 10–59 (87 vs 65%), do farm work (53 vs 26%), be febrile at the time of diagnosis (43 vs 8%) and report no formal education (64 vs 30%) (Table 2).

Overall, index cases were mostly male, between 10 and 39 years of age and worked in farming, particularly in migrant labour activities (especially for the low transmission and high importation group). Among the secondary cases, there were more females, nearly all (119 of 127) reported no measured fever at the time of diagnosis and only 2 reported anti-malarial drug use in the previous 2 weeks. Reported use of a long-lasting insecticide-treated bed net (LLIN) the previous night and having had their house sprayed within the past year varied greatly by village.

Table 3 shows the case investigation operational indicators of villages grouped by transmission pattern. Overall, the mean number of index cases diagnosed per week was 2.1 cases over the study period. The overall mean number of index cases investigated was 1.1 per week, and ranged between 0.8 in low transmission and low importation villages to 7.4 in low transmission and high importation villages. Case investigations were initiated an average of 6.7 days after the index case was diagnosed at the health post, ranging from 0 to 24 days. The mean number of households investigated per index case was 3.9, and the mean number of neighbouring households (within 100-m radius) per index case investigated was 3.3. Case investigations took an average of 2.7 (range 0–33) days to complete; the longest average time observed was in low transmission and low importation villages (6.2 days).

**Table 3:** Operational considerations, by transmission and importation patterns

	Low transmission, low importation villages	Low transmission, high importation villages	High transmission, low importation villages	Total
Mean number (range) of index cases diagnosed per week	1.1 (0–6)	8.2 (0–64)	11.8 (0–53)	2.1 (0–57)
Mean number (range) of index cases investigated per week	0.8 (0–5)	7.4 (0–57)	3.1 (0–16)	1.1 (0–50)
Mean days (range) between index case diagnosis and initiation of investigation	1.9 (0–5)	9.9 (0–24)	0.3 (0–2)	6.7 (0–24)
Mean number (range) of HH per case investigation	5.1 (1–12)	3.6 (1–12)	3.8 (1–12)	3.9 (1–12)
Mean number (range) of neighbouring HH within 100-m radius per index case investigated	4.6 (1–10)	2.9 (1–10)	3.2 (1–10)	3.3 (1–10)
Mean days (range) between initiation and completion of case investigation	6.2 (0–13)	2.0 (0–33)	3.6 (0–10)	2.7 (0–33)

Among the 127 secondary cases infected with *P. falciparum*/mixed, 5 were referred to a health post, 17 were given chloroquine and 105 were given AL, of which 102 (97.1%) received a follow-up visit. Individuals were followed up a mean of 3.8 days (range 3 to 8) after being treated. At the time of the follow-up visit, all individuals had started their treatment. Among the individuals that were visited on day 4 or later (i.e., after the treatment should have been finished), 84% had completed all doses of the treatment. This was verified by checking the blister pack. No adverse events were reported during follow up. Evaluation of parasite clearance during the follow-up visit was conducted in Dehina Sositu and Yeginid Lomi villages, showing that all 11 individuals treated with AL cleared the parasitaemia and had a negative blood smear between 7 and 13 days after administering the first dose.

## Discussion

In 10 villages spanning five different climate and elevation strata (2 villages per strata) in Amhara, Ethiopia, health workers detected 407 clinical *P. falciparum* or mixed index cases during the peak transmission season of 2014–2015. The feasibility of conducting malaria case investigation and FTAT to find additional malaria infections at the home and among nearby neighbouring households for each index case was evaluated. Case investigation was able to be completed for approximately half (54%) of the cases and an additional 127 secondary cases were identified. However, the feasibility of case investigation and the findings varied greatly and pointed to three different groupings of malaria transmission dynamics.

Six of the 10 study villages showed low numbers of cases and very few cases with recent travel history to areas of malaria transmission (low transmission and low importation). Four of these villages are located in the highland region (elevation > 2000 m) whereas two are located between 1000 and 2300 m. The low number of cases (approximately 1 case per week on average) allowed for case investigations to be completed in the households of the index case and their neighbours. The case investigations were typically initiated within 2 days of the index case diagnosis and reached an average of five households. Under these conditions, the index cases represented a clinical case rate of approximately 0.7 per 1000 population and the case investigations found a much higher risk (nearly 20-fold increase) of malaria infection in the index case household members compared to neighbouring households. This demonstrates the very low transmission risk and the highly focal nature of transmission within individual households in these communities. At this low level of transmission with limited importation, case investigation measures may be highly effective in finding and treating the few additional cases in the community.

The second pattern was observed in three villages where the local transmission risk was apparently very low, but many cases were likely imported as nearly 90% had reported travel to a malaria-endemic area within the past month (low transmission and high importation). Occupational data showed that nearly all of the cases, and including 11 of the 15 secondary cases, reported being farmers. From conversations with HEWs, most of these were seasonal migrant labourers returning from work in farms in the western parts of Amhara Region, a lower elevation region known to have higher transmission risk. For these villages, the high number of cases (an average of more than 8 index cases per week) meant that they required extra staff to investigate the cases leading to a delay, such that case investigations began on average more than 9 days after the index case identification. This heightened workload meant that the average number of households visited in the investigation was fewer and only an average of 2 neighbouring households per index case were included in the investigation. Under these conditions, the interval parasite index was approximately 6.6 per 1000 population and additional case finding was not significantly higher in the index case household compared to neighbouring households (1.4 compared to 0.8%, respectively). Of note, in previous years when this level of information on travel was not readily available, these villages would have been designated as having an acute malaria outbreak and may have received aggressive treatment and vector control activities (e.g., extensive insecticide spraying of houses). Given this more detailed information, the risk of local transmission in the village appeared relatively low (only 15 additional cases were identified during the FTAT) and the required priority interventions need to address the source of infections at the distant farms and appropriate care and management for these migrant labourers just prior to or upon their return to their home villages. While case investigation was valuable in that it uncovered the role of exposure in migrant labourers, the case investigation procedures would have been most efficient if targeted only to homes with residents who had recently returned from the farms.

The third pattern of transmission (higher transmission and low importation risk) was observed in one village (Kumar Aftit) that is located in western Amhara at a lower elevation and near the farming areas. This village had a very large number of passively identified cases, averaging more than 11 per week, which overwhelmed the case investigation team such that an average of only 3 index cases could be investigated per week and on average only a quarter of the households that were eligible for inclusion in the investigation were visited. Early in the process, it was recognized by the health team that many of the subsequent index cases were actually from the same neighbourhood as previously investigated index cases. This village had an interval rate of index cases of 62 per 1000 population (tenfold higher than the second pattern seen in the 3 villages noted above) and the investigations identified high number of additional cases in both the index case households and in the neighbouring households (RDT-positivity of 12 and 8%, respectively). Very few of the cases found as index cases or by investigation had a history of travel outside of the area; thus, this high infection burden apparently represents substantial local transmission. This village was clearly above the limit of malaria transmission intensity where case investigation is feasible or likely to be effective in reducing the transmission risk. This village requires additional attention for improved vector control, case management and strengthened systems, including the information systems, to address this higher burden area.

Malaria transmission intensity in Amhara Region is highly linked to geography/elevation differences whereby increased malaria risk is greatest in the areas with more consistent and larger volumes of rainfall that occur in the western Amhara area bordering Sudan, the farming areas. Between June and December each year, there are an estimated 200,000–300,000 migrant workers [10] who travel within Amhara to large agricultural farms. The agricultural season and this travel coincide with the malaria season, ranging from September through to November, with a peak in October. Malaria transmission at these farms is higher, thus migrant workers who are exposed to malaria during the agricultural season often present with symptoms upon their return to their home villages, resulting in the appearance of a false outbreak. Given that migrant workers may serve as the bridge between low and high transmission areas [11], it is critical to recognize and address these ‘outbreaks’ that they bring back to their villages. As re-introduction of malaria through population movement is a threat to elimination of transmission and/or maintaining zero local transmission, more evaluation is needed to understand migration patterns among farm workers in order to tailor more effective malaria control interventions among this mobile population. One possible solution may be to test, and if necessary, treat migrant workers as they leave the farms, or upon returning home, at the end of the agricultural work season.

Majority of both *P. vivax* and *P. falciparum*/mixed secondary cases were afebrile and had no history of fever in the past 24 h. This suggests that access to care among the symptomatic individuals was good and permitted prompt visits, testing and treatment. As a consequence, the remaining infected people would be missed by the passive case detection at health posts; thus, an active investigation process may be a requirement to achieve the elimination goal. Despite the value of test and treat strategies, additional vector control may be warranted as part of the solution for ending transmission in these low transmission areas of Amhara, Ethiopia and searching for persistent vector populations should be explored [12].

The fact that most index cases were investigated and completed in fewer than 3 days, demonstrates that case investigation and reactive FTAT intervention was operationally feasible in low transmission area of Amhara. The reason the number of days to initiate case investigations in Berhan Chora was much longer than in other villages was that SAs were overwhelmed with the large number of index cases among the returning migrant worker population. The study procedures had to be changed: on 1 November, 2014, the radius of investigation was decreased to just include the index case households, especially since the secondary cases detected were near to zero. Nonetheless, the team was able to successfully establish a strong and rapid surveillance system where once an index case was identified, immediate action was taken.

Of note, using the criteria of low versus higher transmission and low *versus* higher importation, none of the study villages exhibited a pattern of both high transmission and high importation. This is not surprising in that higher transmission areas tend to be sources of infection for other areas (exporters of malaria) and imported cases may go relatively unnoticed amidst the high endemicity of the area. Interventions for such areas would most likely begin with reducing the high malaria transmission among the local stable population to reveal the details of importation risks (as was observed in the low transmission and high importation areas).

This study has several limitations. As two intervention villages per each transmission stratum were selected, the 10 intervention villages are not representative of the entire Amhara region, and these findings should not be assumed to be generalizable. Many index cases were not investigated, and as the characteristics or the malaria-risk factors of these index cases were unknown, it is possible that they could be systematically different from index cases that were investigated. Due to changes in the study procedures implemented in Berhan Chora, secondary cases in neighbouring households were likely missed, thus the estimates for this village are conservative and should be interpreted with caution. Identification of index cases was limited to health posts, and it is possible that cases seen at other health facilities (health centres or nearby health facilities) but outside of the catchment area, may not have been captured to initiate case investigations. This intervention followed a MTAT intervention that occurred at the outset of the rains in six of the 10 villages (3 in low transmission and low importation; 2 in low transmission and high importation; and one village with high transmission and low importation) and this could have influenced the findings, which might not reflect the situation that would be encountered when initiating case investigation in villages without a previous MTAT. Further review of possible effects on index and secondary cases, showed that MTAT did not influence the subsequent findings of FTAT later in the transmission season in one village with high transmission and low importation. RDTs that differentiate between *P. falciparum* infections and non-falciparum *Plasmodium* infections to diagnose and subsequently treat malaria infections and to categorize index cases as either *P. falciparum* or mixed infections were used; we are not able to separately categorize and characterize all of the *P. vivax* index infections. Also, the RDTs may have missed infections with lower antigen levels that could have been detected by more sensitive assays (PCR) [13, 14], or could provide false positives due to lingering HRP2 or non-Plasmodium infectious agents and immunological factors [15] . Results from previous work in these areas in Amhara Region with RDTs, microscopy and serologic assays suggest that a portion of the *P. vivax* infections may have been missed [16]. Further evaluation of the use of more sensitive RDTs may identify additional infections to be cleared as part of efforts to reduce malaria transmission. Lastly, we categorized villages by low or high importation based on travel history, assuming cases with travel history are likely imported. However, genotyping was not conducted, thus misclassification will certainly have occured and importation might be overestimated. Additionally, given the temporal and spatial movement of migrant workers within Amhara, it is possible that ascertainment of travel history was not precise, hence some villages could be classified into different patterns and results may not be generalizable.

## Conclusion

This study suggests that case investigation with reactive FTAT is operationally feasible when case numbers are sufficiently low to permit efficient investigation (perhaps fewer than 5 per week at a health facility) and where health staff has training and capacity. The benefits of this intervention when transmission is very low include the ability to: identify malaria infections in the community that would otherwise be missed; determine whether cases were likely indigenous or imported (using travel history as a proxy); examine where malaria cases cluster geographically; characterize the demographic profile and malaria risk factors of this population; and, inform the development and modification of intervention strategies that can support malaria elimination. Moreover, the distinct patterns of malaria transmission observed in this study warrant intervention strategies that specifically target the higher transmission areas and the mobile population that acquires infection in these higher transmission areas and brings it to areas that are approaching elimination.

## Additional file

**Additional file 1.** Study profile of index cases and FTAT investigation, by village.

## Abbreviations

ANRSHB: Amhara National Regional State Health Bureau
CI: confidence interval
FMOH: Federal Ministry of Health
IRS: indoor residual spraying
FTAT: focal test and treat
MACEPA: Malaria Control and Elimination Partnership in Africa
ODK: Open Data Kit
RDT: rapid diagnostic test
