# Supplementary material for: Malaria case investigation with reactive focal testing and treatment: operational feasibility and lessons learned from low and moderate transmission areas in Amhara Region, Ethiopia
Source: Malar J. 2018 Dec 4;17:449. doi: 10.1186/s12936-018-2587-8 (PMC6278130; doi:10.1186/s12936-018-2587-8)
Supplement: Supplementary file 1 — Additional file 1. Study profile of index cases and FTAT investigation, by village. [file 12936_2018_2587_MOESM1_ESM.docx]

**Additional files**

**Additional file 1** Study profile of index cases and FTAT investigation, by village

|  | **Low transmission,**  **low importation villages** | | | | | | **Low transmission,**  **high importation villages** | | | **High transmission, low importation village** |  |
| --- | --- | --- | --- | --- | --- | --- | --- | --- | --- | --- | --- |
|  | **Ancharo** | **Choresa** | **Dehina**  **Sositu** | **Hardibo** | **Ketie** | **Zengoba Denguma** | **Berhan**  **Chora*** | **Enashenifalen** | **Yeginid**  **Lomi** | **Kumar**  **Aftit*** | **Total** |
| **2015 Population estimates** | **4,702** | **4,992** | **9,552** | **3,941** | **6,271** | **4,836** | **8,404** | **10,203** | **5,077** | **3,666** | **61,644** |
| **Index cases** |  |  |  |  |  |  |  |  |  |  |  |
| Passively detected *P. falciparum*/mixed, n | 1 | 3 | 9 | 2 | 3 | 5 | 134 | 14 | 8 | 228 | 407 |
| Investigated, n (%) | 1 (100) | 2 (66.7) | 9 (100.0) | 1 (50.0) | 3 (100.0) | 2 (40.0) | 119 (88.8) | 14 (100.0) | 8 (100.0) | 61 (26.8) | 220 (54.1) |
| Investigated cases with travel history in past 30 days, n (%) | 0 (0) | 0 (0) | 1 (11.1) | 0 (0) | 0 (0) | 1 (50.0) | 112 (95.0) | 9 (64.3) | 5 (62.5) | 0 (0) | 128 (58.2) |
| Investigated cases with no travel history in past 30 days, n (%) | 1 (100.0) | 2 (100.0) | 8 (88.8) | 1 (100.0) | 3 (100.0) | 1 (50.0) | 7 (5.0) | 5 (35.7) | 3 (37.5) | 61 (100.0) | 92 (41.8) |
| Interval parasite index (per 1,000 population) | 0.2 | 0.6 | 0.9 | 0.5 | 0.5 | 1.0 | 16.0 | 1.4 | 1.6 | 62.2 | 6.6 |
| Interval parasite index for travellers | 0.0 | 0.0 | 0.1 | 0.0 | 0.0 | 0.2 | 13.3 | 0.9 | 1.0 | 0.0 | 2.1 |
| Interval parasite index for non-travellers | 0.2 | 0.6 | 0.8 | 0.5 | 0.5 | 0.6 | 0.95 | 0.5 | 0.6 | 62.2 | 4.3 |
| **FTAT investigation** |  |  |  |  |  |  |  |  |  |  |  |
| **Index case HH** |  |  |  |  |  |  |  |  |  |  |  |
| Individuals in HHs, n  (excluding index cases) | 2 | 10 | 39 | 3 | 13 | 7 | 441 | 59 | 29 | 270 | 873 |
| Individuals tested, n (%) | 2 (100.0) | 9 (90.0) | 33 (84.6) | 3 (100.0) | 13 (100.0) | 6 (85.7) | 418 (94.8) | 56 (94.9) | 29 (100.0) | 203 (75.2) | 772 (88.4) |
| RDT positivity, n (%) | 0 (0) | 1 (11.1) | 1 (3.0) | 0 (0) | 1 (7.7) | 2 (33.3) | 5 (1.2) | 0 (0) | 2 (6.9) | 25 (12.3) | 37 (4.8) |
|  |  |  |  |  |  |  |  |  |  |  |  |
| **Neighbouring HH** |  |  |  |  |  |  |  |  |  |  |  |
| HHs visited, n | 10 | 20 | 58 | 1 | 19 | 4 | 118 | 83 | 53 | 328 | 694 |
| Individuals in HHs, n | 44 | 96 | 291 | 4 | 75 | 19 | 512 | 393 | 208 | 1341 | 2,983 |
| Individuals tested, n (%) | 38 (86.4) | 77 (80.2) | 257 (88.3) | 4 (100.0) | 72 (100.0) | 19 (100.0) | 451 (88.1) | 361 (91.9) | 190 (91.3) | 1,002 (74.7) | 2,471 (82.8) |
| RDT positivity, n (%) | 0 (0) | 0 (0) ^**^ | 1 (0.4) | 0 (0) | 1 (1.4) | 0 (0) ^**^ | 5 (1.1) | 1 (0.3) | 2 (1.1) ^**^ | 80 (8.0)^**^ | 90 (3.6) |
|  |  |  |  |  |  |  |  |  |  |  |  |

*In Berhan Chora and Kumer Aftit, the FTAT procedures were modified on 1 November, 2014 because it was not possible to complete case investigations for every index case. If the index case had a travel history, only the index case household was visited for the case investigation rather than the index case household plus up to 10 neighbouring households.

**Significant *P* value comparing RDT positivity in Index HH and Neighbouring HH (*P*<0.05)

FTAT: Focal test and treat; HH: household.
